# Evaluating the Effect of Knowledge, Attitude, and Practice on Self-Management in Type 2 Diabetic Patients on Dialysis

**DOI:** 10.1155/2016/3730875

**Published:** 2016-07-10

**Authors:** Shima Ghannadi, Atieh Amouzegar, Parisa Amiri, Ronak Karbalaeifar, Zhale Tahmasebinejad, Sara Kazempour-Ardebili

**Affiliations:** ^1^Endocrine Research Center, Research Institute for Endocrine Sciences, Shahid Beheshti University of Medical Sciences, Tehran, Iran; ^2^Research Center for Social Determinants of Endocrine Health, Research Institute for Endocrine Sciences, Shahid Beheshti University of Medical Sciences, Tehran, Iran; ^3^Statistics Unit, Research Institute for Endocrine Sciences, Shahid Beheshti University of Medical Sciences, Tehran, Iran; ^4^Prevention of Metabolic Disorders Research Center, Research Institute for Endocrine Sciences, Shahid Beheshti University of Medical Sciences, Tehran, Iran

## Abstract

*Background.* Type 2 diabetes is an increasingly common condition with several preventable microvascular complications such as kidney damage. Nephropathy is expensive to manage, especially as hospital dialysis treatment. Improving patients' knowledge, attitude, and practice (KAP) toward their condition can achieve better control, delay complications, and improve their quality of life. This study evaluated the KAP and self-care behaviors of diabetic patients on dialysis and variables that affect it.* Methods.* This cross-sectional study was conducted at Shahid Beheshti academic hospitals of Tehran, Iran. Face-to-face interviews were held to fill five validated questionnaires: three evaluating KAP, one evaluating self-management, and one evaluating quality of life.* Result.* 117 diabetic patients on hemodialysis (42 females) with mean (SD) age of 68.70 ± 9.26 years were enrolled in the survey. The scores for patient's KAP, self-care, and quality of life were 59.90 ± 11.23, 44.27 ± 8.35, 45.06 ± 12.87, 46.21 ± 10.23, and 26.85 ± 13.23, respectively. There was significant negative correlation between patients' knowledge and attitude with their glycosylated hemoglobin level and their fasting blood sugar. There was significant correlation between patients' knowledge and practice with their self-care activities.* Conclusion.* The present study suggests that patients' KAP scores have a practical effect upon self-care behavior. This highlights the needs for effective diabetes education programs in developing countries like Iran.

## 1. Introduction

Type 2 diabetes (formerly called non-insulin-dependent or adult-onset) results from the body's ineffective use of insulin. Type 2 diabetes comprises 90% of people with diabetes around the world [1]. 347 million people worldwide have diabetes [2]. There is an emerging global epidemic of diabetes that can be traced back to rapid increases in overweight, including obesity and physical inactivity. In 2012 diabetes was the direct cause of 1.5 million deaths; more than 80% of diabetes deaths occur in low- and middle-income countries [3]. The prevalence of DM is high among populations in the Middle East and Persian Gulf countries, and patients often lack the knowledge and skills to self-manage their condition [4–6]. The prevalence of type 2 DM in Iran has grown over the past decade [7], and the age of onset of diabetes in Iran is about 10 to 15 years less than the world standard [8]. It is therefore expected that the true prevalence of diabetes is more than what has been previously reported in Iran. This high prevalence rate of diabetes is mainly due to sedentary life style, urbanization, lack of knowledge about the disease, low literacy rate, and socioeconomic status [9] that make diabetes awareness and self-management major challenges in Iran.

DM is an important and growing health problem in both developed and developing countries as it requires life-long medical and life style adjustment [10]. It causes disability and premature mortality and increases demands on healthcare facilities [11]. One of the most important complications of diabetes is nephropathy. Type 2 diabetes is the leading cause of incident and prevalent chronic kidney disease (CKD), accounting for about 30% to 40% of CKD and up to 45% of end-stage renal disease (ESRD) [12, 13]. In the past 2 decades, there has been a continual increase in the incidence of ESRD among patients with diabetes, predominantly those with type 2 diabetes [14]. Diabetes may cause acute and chronic complications [15] if serious consideration is not taken in early stages of development. These complications can affect the quality of life of patients [16, 17] and may lead to increased morbidity and mortality [18]. The most common complications of diabetes have imposed high costs on both the individual and society. Onset of complications, especially if it is combined with large and small vessel disease, can lead to reduced quality of life [19]. Prevention, pathogenesis, and mortality due to theses complications are considered major healthcare issues in the world [20], and that is why considerable attention has shifted to the investment for diabetes control [21].

Managing DM and its complications is very costly. Many studies have shown that control of hyperglycemia in diabetic patients can prevent or reduce the risks of diabetic complication [22]. Better glycemic management of DM requires not only the prescription of appropriate nutritional and pharmacological regime by the physician but also intensive education of the patient [23]. Large studies about this relationship have used measurements such as HbA1c, lipids, KAP, and self-efficacy as the index of diabetes management [24, 25]. The use of self-management programs in chronic disease is relatively well known, and some of these programs are beginning to show success [26]. However, there is little evidence to suggest that programs specifically for diabetic renal disease have been developed or tested.

Very few studies [27, 28] have been conducted in Iran to evaluate the level of awareness, attitudes, and practices among type 2 diabetic patients, and we found no study carried out specifically on type 2 diabetic patient receiving hemodialysis. Therefore, this study evaluated the knowledge, attitude, and practices of diabetes patients on hemodialysis and variables that affect their KAP scores.

## 2. Methods

This study was a cross-sectional study. Type 2 diabetic patients who are receiving hemodialysis in Shahid Beheshti academic hospitals of Tehran, the capital of Iran, were enrolled in the study during period of April to June 2014. This study was carried out in hemodialysis wards of five academic hospitals of Tehran (Shohadaye Tajrish, Modarres, Emam hosein, and Labbafi Nejad). These hospitals provide healthcare services to patients of all regions of Tehran.

Patients not interested in taking part in the study, those lacking compliance for face-to-face interview, and inpatients were excluded. By taking the lowest correlation ratio, a sample size of 113 was calculated. This study managed to enroll a total of 117 patients.

An expert nurse measured systolic and diastolic blood pressure and the body mass index (BMI). Patients receiving antihypertensive medication or those with BP >124/84 mmHg were considered as hypertensive. Known cases of dyslipidemia (high LDL-cholesterol, low HDL-cholesterol, and/or high triglycerides) receiving medication were considered as dyslipidemia. Nonproliferative and proliferative retinopathy were both considered as retinopathy. Weakness, numbness, and pain in hands or feet were considered as neuropathy.

A KAP (Knowledge, Attitude, and practice) questionnaire which was locally developed and validated was used [29]. This questionnaire consisted of 4 parts including demographic information, knowledge (10 questions), attitude (10 questions), and practice (11 questions). Questions of the knowledge and practice parts were multiple choice questions with 0-1 and 0–4 scores, based on number of correct choices. Questions of the attitude part were −2 to +2 (strongly agree, agree, no idea, disagree, and strongly disagree).

The summary of diabetes self-care activities (SDSCA) measure is a brief self-report questionnaire of diabetes self-management that includes 25 items assessing the seven aspects of the diabetes regimen: general diet, specific diet, exercise, blood–glucose testing, medications, foot care, and cigarette smoking. Response options range from 0 to 7 to correspond to the number of days in a week [30].

The audit of diabetes-dependent quality of life (ADDQoL) questionnaire was originally designed in 1994 and has been widely applied in many countries and is viewed as a particular and useful scale of useful diabetes-specific tool. This questionnaire consists of 19 questions. Each question is +1 (positive effect) to −3 (very negative effect) scores. This questionnaire has also previously been validated [31, 32].

A total of 117 patients were enrolled in the study. Their demographic details and laboratory data were noted in demographic information form. We had face-to-face interview with all the patients to fill the five questionnaires.

Data were analyzed using statistics package for social science (SPSS) v.21 for windows. All the scores were scaled from zero to one hundred. All continuous Gaussian data are expressed as mean (SD) and non-Gaussian data as median and interquartile range; categorical variables are expressed as No (%). Quantitative variables were checked for normality using the Kolmogorov-Smirnoff test. Differences of continuous variables between groups were analyzed using independent *t*-test, ANOVA, and Tukey's Post Hoc tests for Gaussian data and Mann-Whitney for non-Gaussian. Additionally, Chi-square test was used for comparison of categorical variables which were expressed as percentages. Moreover, the correlation between variables was tested using Pearson's and Spearman's correlation considering the Gaussian and non-Gaussian distribution of variables, respectively. *p* values less than 0.05 were considered as significant.

The ethical clearance of this study was obtained from the ethical committee of research in Shahid Beheshti University of Medical Sciences, Tehran, Iran. The details of study were explained for patients and written informed consent was obtained from all patients. Enrollment in the study did not disrupt the patients' treatment process. All patients' information was kept secure and anonymous.

## 3. Result

In total, 117 diabetic patients on hemodialysis (42 females and 75 males) with the age of 50–88 years, mean (SD) 68.70 (9.26) years, and mean (SD) BMI of 25.07 (4.02) were enrolled in the survey. Subjects were evenly distributed in terms of their geographical area of residence, which is important as it covers all regions of Tehran with different socioeconomic levels.

The mean (SD) of duration of the disease among type 2 diabetes mellitus patients on dialysis was 14.23 (7.29) years. 93 (79.5%) subjects were treated with insulin and the others were not treated with any drugs for their diabetes. Subjects' baseline characteristics are shown in Table 1.

The results showed the average ± SD score of knowledge: 59.90 ± 11.23, for attitude: 44.27 ± 8.35, for practice: 45.06 ± 12.87, for self-care: 46.21 ± 10.23, and quality of life: 26.85 ± 13.23. All data are shown in Appendix Tables 1–4 (in Supplementary Material available online at http://dx.doi.org/10.1155/2016/3730875).

Gender, marital status, and area of residence had no statistically significant association with KAP or self-care scores. Self-care score was lower among those with disease duration of more than 10 years (49.8 versus 56.4, *p* = 0.035). Both practice and self-care scores were higher among dialysis patients who were on insulin compared to those not on insulin (or any other glucose-lowering treatment) (46.4 versus 39.8, *p* = 0.023 and 55.6 versus 33.5, *p* < 0.001, resp.). Patients with a positive family history of diabetes had lower self-care scores compared to those with no family history (49.8 versus 59.1, *p* = 0.034). Higher education level was associated with significantly higher knowledge, practice, and self-care scores, but not with improved attitude.

Among risk factors of disease, hypertension and smoking were associated with lower self-care scores and current smoking was associated with poor attitude. Subjects with a positive history of diabetic foot had better practice scores compared to those with no such history, which may indicate increased awareness after the development of the complication (Table 2).

Increased age was correlated with lower knowledge scores. Higher FPG and HbA1c were both significantly correlated with lower knowledge and attitude scores. Higher knowledge score was significantly correlated with higher attitude, practice, and self-care scores and higher practice score was also significantly associated with higher self-care score. Improved quality of life had a significant correlation with improved attitude (Table 3). There was also a significant correlation between self-care's subscales such as exercise, glucometer BS, and foot care scores with patients' knowledge and practice levels (Table 4).

## 4. Discussion

This study shows a low level of knowledge, unfavorable attitude, and moderate practice scores among our patient population of type 2 diabetes patients undergoing hemodialysis. We also observed a positive correlation between knowledge scores and attitude, practice, and self-care as well as attitude with quality of life and practice with self-care. Furthermore, indices of poor glycemic control, that is, high FPG and HbA1c levels, were significantly correlated with lower knowledge and attitude scores. The relatively low baseline HbA1c level in this population of patients with advanced diabetes is expected as HbA1c levels are usually underestimated in subjects on dialysis [33].

We found that the mean score for knowledge was 59.90 ± 11.23 of the maximum 86.36 score possible. The low level of knowledge of disease in type 2 diabetes mellitus patients on hemodialysis may reflect the low emphasis on patient education. In addition, the reason for a lower knowledge level in this study may be partly explained by the sample involving patients on dialysis, who are generally older and had lower education level; patient's age was between 50 and 88 years, mean (SD) 68.70 ± 9.26 years, and most of them, 84 (71.7%), had primary educational degree. While Coates and Boore observed a higher mean knowledge score among their diabetic patients compared to our population, declaring that knowledge is only one of several variables that influence metabolic control [34]. Upadhyay and colleagues observed lower knowledge scores than the present study [35] among their newly diagnosed patient population.

We found that the majority of the diabetic patients in our survey had unfavorable attitude (mean score of 44.27 ± 8.35 with a maximum score of 62.50). We suggest that this can be due to a low knowledge level among the patients as well as that of their healthcare team on how to improve their patients' attitude. Kozier and Lea Erb found that nurses had an important role for helping patients in changing their attitude and they can help in overcoming the barriers and support positive activities [36].

In our study, patients' mean practice score was low (45.06 ± 12.87 with a maximum possible score of 72.73). This can also be due to the subjects' low education as well as their poor attitude towards their condition. Kennedy et al. reported that education on self-care had positive influence on patients' practice scores in their survey of Mexican American women [37].

In the study by Niroomand et al., patients' knowledge, attitude, and practice (KAP) were significantly higher than the present study which can be attributed to differences in study population, as they had evaluated diabetic patients without complications while we looked at diabetic patients receiving hemodialysis in the present study [29].

While we observed a positive correlation between improved glycemic control as shown by lower FPG and HbA1c levels and higher knowledge and attitude score, a study by Mohammadi and colleagues showed no significant association was observed between KAP score and glycemic control [38]. Their result was reflected by a similar study carried out among Malaysian diabetes patients [39]. While our observation seems more intuitive, as improved knowledge of disease and a better attitude should improve glycemic control, the differences in the results of these studies with our results may be explained by the differences in our specific population and the fact that they had all developed a serious complication.

We have observed a significant association between patients' knowledge and practice with their self-management behavior, which seems to be logical. However, Toobert et al. showed that diabetic patients' knowledge and practice were not related significantly to their self-management [30]. Again, this difference may be due to our specific population who consisted of elderly, poorly educated subjects with ESRD.

In contrast with the results from our survey, some studies have shown that there is no correlation between increased knowledge and metabolic control (HbA1c and BS); Beggan et al. noted that HbA1c as an indicator of metabolic control was not correlated significantly with knowledge [40] and Germer et al. found no correlation between the level of knowledge and the level of control as measured by a random blood glucose or HbA1c [41].

It is well established that patient contributions are very important for better management of diabetes [42]. Lack of knowledge of diabetes care among patients can have adverse effects on their capabilities to control diabetes. CKD has been indicated as one of the most common complications of diabetes by 72% of our subjects. Awareness of measures to identify primary complications of diabetes, such as checking proteinuria and blood pressure monitoring, was poor, which reveals the need for effective diabetes education programs in developing countries like Iran, as it has been stated that knowledge regarding diabetes among the general population as well as those with diabetes is still insufficient in Iran and a good diabetes education program is greatly required [43]. There are few appropriate diabetes education programs in most governmental hospitals and the existing programs are weak.

This study also has some limitations; it was limited to type 2 diabetic patients receiving hemodialysis at academic hospitals and does not include the private sector. Also, all the scores of knowledge, attitude, practice, self-care, and quality of life were scored from 0 to 100 to be comparable with other literatures. As the attitude and quality of life had negative scores in the questionnaire, we know that attitude scores more than 60 and quality of life scores more than 80 are considered positive.

## 5. Conclusion

This study has measured the level of knowledge, attitude, and practice as well as self-care and quality of life among a diabetic population receiving hemodialysis. We have observed a low level of knowledge and attitude that is correlated with subjects' practice scores as well as their glycemic control. We have also observed a positive correlation between attitude scores and quality of life. It can therefore be deducted that investing in proper patient education that increases knowledge and awareness improves attitude and practice and translates to self-care and better quality of life is an imperative undertaking through all the stages of diabetes.

## Supplementary Material

All data about the results of knowledge, attitude, practice and self-care is given in the Supplementary Material by details.

## Figures and Tables

**Table 1:** Description of baseline characteristics of patients.

Variable	Male (*N* = 75)	Female (*N* = 42)	Total (*N* = 117)	*p*
Age, years	68.92 ± 9.79	68.31 ± 8.33	68.70 ± 9.26	0.728
Marital status, number (%)				0.077
Married	69 (92.0)	34 (81.0)	103 (88.0)	
Not married	6 (8.0)	8 (19.0)	14 (22.0)	
Degree, number (%)				<0.001
Primary	44 (58.7)	40 (95.2)	84 (71.7)	
Secondary	21 (28.0)	2 (4.8)	23 (19.7)	
Higher	10 (13.3)	0 (0.0)	10 (8.5)	
Job, number (%)				<0.001
Employed	13 (17.3)	0 (0.0)	13 (11.1)	
Unemployed	26 (34.7)	42 (100.0)	68 (58.1)	
Retired	36 (48.0)	0 (0.0)	36 (30.8)	
Address, number (%)				0.061
North	19 (25.3)	11 (26.2)	30 (25.6)	
East	24 (32.0)	5 (11.9)	29 (24.8)	
West	15 (20.0)	9 (21.4)	24 (20.5)	
South	17 (22.7)	17 (40.5)	34 (29.1)	
Disease duration, years				0.78
<10	14 (18.7)	7 (16.7)	21 (17.9)	
>10	61 (81.3)	35 (83.3)	96 (82.1)	
Treatment methods, number (%)				0.76
Insulin	59 (78.7)	34 (81.0)	93 (79.5)	
Without treatment	16 (21.3)	8 (19.0)	24 (20.5)	
Risk factors, number (%)				
Hypertension	52 (69.3)	23 (54.8)	75 (64.1)	0.115
Dyslipidemia	29 (38.7)	22 (52.4)	51 (43.6)	0.151
Current smoking	11 (14.7)	0 (0.0)	11 (9.4)	0.009
Complications, number (%)				
Nephropathy	75 (100.0)	42 (100.0)	117 (100.0)	·
Retinopathy	75 (100.0)	42 (100.0)	117 (100.0)	·
Neuropathy	75 (100.0)	42 (100.0)	117 (100.0)	·
Diabetic foot	30 (40.0)	12 (28.6)	42 (35.9)	0.216
Family history, number (%)				0.274
Positive	58 (77.3)	36 (85.7)	94 (80.3)	
Negative	17 (22.7)	6 (14.3)	23 (19.7)	
Smoking, number (%)				<0.001
Negative	64 (85.3)	42 (100.0)	106 (90.6)	
Positive	11 (14.7)	0 (0.0)	11 (9.4)	
Systolic blood pressure, mmHg	127.00 ± 18.97	124.62 ± 20.13	126.15 ± 19.34	0.530
Diastolic blood pressure, mmHg	97.52 ± 8.97	76.19 ± 10.40	78.32 ± 9.60	0.072
BMI, kg/m^2^	24.29 ± 3.53	26.47 ± 4.48	25.07 ± 4.02	0.008
FPG, mg/dL	157.01 ± 72.52	165.69 ± 66.19	160.12 ± 70.15	0.523
HbA1c, percent	6.91 ± 1.15	7.23 ± 1.15	7.02 ± 1.16	0.152

**Table 2:** Patients' knowledge, attitude, practice, and self-care scores correlations.

Variable	Knowledge	Attitude	Practice	Self-care
Gender				
Male	61.15 ± 10.93	43.40 ± 8.66	46.66 ± 13.80	48.00 ± 10.83
Female	57.68 ± 11.55	45.83 ± 7.60	42.20 ± 10.59	43.00 ± 8.23
*p* value	*p* = 0.110	*p* = 0.131	*p* = 0.072	*p* = 0.270
Disease duration				
<10 years	58.87 ± 9.58	45.47 ± 8.57	49.35 ± 9.77	56.37 ± 13.17
>10 years	60.13 ± 11.60	44.01 ± 8.32	44.12 ± 13.32	49.88 ± 12.46
*p* value	*p* = 0.644	*p* = 0.469	*p* = 0.092	*p* = 0.035
Medication				
Insulin	60.21 ± 11.10	45.00 ± 8.01	46.43 ± 12.09	55.56 ± 9.86
Without treatment	58.71 ± 11.90	41.45 ± 9.17	39.77 ± 14.63	33.54 ± 5.71
*p* value	*p* = 0.561	*p* = 0.064	*p* = 0.023	*p* < 0.001
Family history				
Yes	60.20 ± 11.42	44.73 ± 7.82	44.87 ± 13.11	49.80 ± 13.14
No	58.69 ± 10.60	42.39 ± 10.21	45.84 ± 12.09	59.10 ± 9.89
*p* value	*p* = 0.566	*p* = 0.229	*p* = 0.746	*p* = 0.034
Marital status				
Married	60.15 ± 11.52	44.19 ± 8.30	45.89 ± 13.06	51.64 ± 12.75
Not married	55.15 ± 7.80	44.82 ± 9.01	38.96 ± 9.71	46.61 ± 12.55
*p* value	*p* = 0.120	*p* = 0.795	*p* = 0.058	*p* = 0.168
Degree				
Primary	58.27 ± 10.68	44.79 ± 8.22	41.99 ± 11.71	49.01 ± 11.79
Secondary	62.64 ± 11.62	43.36 ± 9.64	53.35 ± 12.35	54.84 ± 14.87
Higher	67.27 ± 11.89	42.00 ± 6.10	51.81 ± 13.58	59.40 ± 11.40
*p* value	*p* = 0.023	*p* = 0.517	*p* < 0.001	*p* = 0.014
Job				
Employed	63.11 ± 10.70	44.03 ± 8.63	50.34 ± 15.04	55.29 ± 13.94
Unemployed	57.60 ± 10.92	44.09 ± 8.65	42.64 ± 11.32	49.22 ± 11.04
Retired	61.11 ± 11.62	44.72 ± 7.87	44.94 ± 12.66	50.76 ± 13.99
*p* value	*p* = 0.088	*p* = 0.929	*p* = 0.041	*p* = 0.135
Address				
North	60.30 ± 10.60	44.83 ± 6.72	43.93 ± 11.47	49.31 ± 12.94
East	61.75 ± 11.62	44.31 ± 7.06	46.39 ± 13.81	51.74 ± 13.84
West	58.90 ± 12.02	46.97 ± 6.75	48.10 ± 13.79	56.38 ± 12.58
South	58.68 ± 11.15	41.83 ± 10.92	42.78 ± 12.56	48.20 ± 11.05
*p* value	*p* = 0.708	*p* = 0.182	*p* = 0.404	*p* = 0.088

Risk factors				
Hypertension				
Positive	59.93 ± 11.80	44.06 ± 7.83	43.03 ± 12.71	49.31 ± 13.46
Negative	59.84 ± 10.28	44.64 ± 9.28	48.70 ± 12.51	54.13 ± 10.95
*p* value	*p* = 0.967	*p* = 0.722	*p* = 0.022	*p* = 0.038
Dyslipidemia				
Positive	61.22 ± 11.96	45.09 ± 8.20	46.70 ± 13.23	51.97 ± 11.67
Negative	58.88 ± 10.62	43.63 ± 8.47	43.80 ± 12.54	50.32 ± 13.62
*p* value	*p* = 0.265	*p* = 0.350	*p* = 0.299	*p* = 0.492
Current smoking				
Positive	57.85 ± 12.21	49.09 ± 15.40	40.28 ± 12.58	29.54 ± 15.65
Negative	60.12 ± 11.17	43.77 ± 8.32	45.54 ± 12.57	52.16 ± 12.33
*p* value	*p* = 0.526	*p* = 0.044	*p* = 0.128	*p* = 0.003

**Table 3:** Correlation of patients' knowledge, attitude, practice, and self-care with confounders.

Variable	Knowledge	Attitude	Practice	Self-care
Age	*r* = −0.284	*r* = −0.060	*r* = −0.113	*r* = −0.169
	*p* = 0.002	*p* = 0.523	*p* = 0.227	*p* = 0.069

FPG	*r* = −0.277	*r* = −0.212	*r* = −0.118	*r* = −0.122
	*p* = 0.003	*p* = 0.022	*p* = 0.203	*p* = 0.191

HbA1c	*r* = −0.438	*r* = −0.253	*r* = −0.141	*r* = −0.077
	*p* < 0.001	*p* = 0.006	*p* = 0.129	*p* = 0.410

BMI	*r* = 0.004	*r* = −0.096	*r* = 0.119	*r* = 0.143
	*p* = 0.963	*p* = 0.301	*p* = 0.202	*p* = 0.125

Quality of life	*r* = 0.119	*r* = 0.250	*r* = 0.012	*r* = 0.006
	*p* = 0.201	*p* = 0.006	*p* = 0.897	*p* = 0.953

*K* score		*r* = 0.228	*r* = 0.386	*r* = 0.253
		*p* = 0.013	*p* < 0.001	*p* = 0.006

*A* score			*r* = −0.148	*r* = −0.028
			*p* = 0.110	*p* = 0.766

*P* score				*r* = 0.597
				*p* < 0.001

**Table 4:** Correlation of patients' knowledge, attitude, practice, and quality of life with self-care subscales.

Variable (self-care)	Knowledge	Attitude	Practice	Quality of life
Diet	*r* = 0.022	*r* = 0.049	*r* = 0.122	*r* = 0.299
	*p* = 0.814	*p* = 0.597	*p* = 0.190	*p* = 0.001

Exercise	*r* = 0.217	*r* = −0.137	*r* = 0.347	*r* = 0.470
	*p* = 0.019	*p* = 0.142	*p* < 0.001	*p* < 0.001

BS glucometer	*r* = 0.269	*r* = 0.016	*r* = 0.608	*r* = 0.725
	*p* = 0.003	*p* = 0.886	*p* < 0.001	*p* < 0.001

Foot care	*r* = 0.265	*r* = −0.144	*r* = 0.519	*r* = 0.424
	*p* = 0.004	*p* = 0.122	*p* < 0.001	*p* < 0.001
