# Supplementary material for: Evaluating the Effect of Knowledge, Attitude, and Practice on Self-Management in Type 2 Diabetic Patients on Dialysis
Source: J Diabetes Res. 2016 Jul 10;2016:3730875. doi: 10.1155/2016/3730875 (PMC4958437; doi:10.1155/2016/3730875)
Supplement: Supplementary file 1 — All data about the results of knowledge, attitude, practice and self-care is given in the Supplementary Material by details. [file 3730875.f1.docx]

**Appendix:**

1. **Table1.** Description of Patients’ Knowledge Score in Detail

| **Knowledge** | **Male** | **Female** | **Total Score** |
| --- | --- | --- | --- |
| K1. What are diabetes symptoms? (0 - 4) | 1.74 ± 0.63 | 1.54 ± 0.59 | 1.67 ± 0.62 |
| K2. What is the effect of exercise on glucose controlling? (0 - 1) | 0.68 ± 0.46 | 0.52 ± 0.50 | 0.62 ± 0.48 |
| K3. Is dietary intervention necessary in controlling glucose? (0 - 1) | 0.98 ± 0.11 | 1.00 ± 0.00 | 0.99 ± 0.09 |
| K4. What is necessary for controlling diabetes? (0 - 4) | 2.82 ± 0.64 | 2.95 ± 0.69 | 2.87 ± 0.66 |
| K5. What is suitable blood pressure for a diabetic patient? (0 - 1) | 0.46 ± 0.50 | 0.42 ± 0.50 | 0.45 ± 0.49 |
| K6. Which index is suitable for awareness about diabetes control in past months? (0 - 1) | 0.02 ± 0.16 | 0.00 ± 0.00 | 0.01 ± 0.13 |
| K7. Which one is the normal blood glucose in a healthy person? (0 - 1) | 0.42 ± 0.49 | 0.30 ± 0.46 | 0.38 ± 0.48 |
| K8. Which one could cause type 2 diabetes? (0 - 4) | 2.69 ± 0.71 | 2.59 ± 0.79 | 2.65 ± 0.74 |
| K9. Which one is the correct foot care in a diabetic person? (0 - 1) | 0.53 ± 0.50 | 0.30 ± 0.46 | 0.45 ± 0.49 |
| K10. What is the effect of diabetes on eyes? (0 - 4) | 3.06 ± 0.55 | 3.02 ± 0.41 | 3.05 ± 0.50 |
| Total knowledge score (0-100) | 61.15 ± 10.93 | 57.68 ± 11.55 | 59.90 ± 11.23 |

1. **Table2.** Description of Patients’ attitude Score in Detail

| **Attitude** | **Male** | **Female** | **Total Score** |
| --- | --- | --- | --- |
| A1. Diabetes mellitus is treatable. | 0.44 ± 1.08 | 0.26 ± 1.07 | 0.37 ± 1.06 |
| A2. Diabetes mellitus is treatable with dietary and exercise. | 0.53 ± 0.96 | 0.69 ± 0.71 | 0.58 ± 0.88 |
| A3. Medication can be discontinued in case of increasing blood glucose and symptoms release. | 0.44 ± 1.00 | 0.40 ± 0.93 | 0.42 ± 0.97 |
| A4. Diabetes reduces life expectancy. | - 1.02 ± 0.61 | - 1.09 ± 0.57 | - 1.05 ± 0.59 |
| A5. Herbal medications have less complication than physicians’ medications. | - 0.18 ± 0.76 | - 0.16 ± 1.05 | - 0.17 ± 0.87 |
| A6. Lipid and blood pressure control is necessary in diabetic patients. | - 1.16 ± 0.49 | - 1.02 ± 0.41 | -1.11 ± 0.46 |
| A7. Regular exercise helps controlling diabetes. | - 0.77 ± 0.68 | - 0.57 ± 0.70 | - 0.70 ± 0.69 |
| A8. Initiating insulin exacerbates diabetes and its complications. | 0.72 ± 0.90 | 1.09 ± 0.75 | 0.85 ± 0.87 |
| A9. Proper diabetes treatment could blockage renal failure and blindness. | - 1.17 ± 0.66 | - 1.07 ± 0.60 | - 1.13 ± 0.64 |
| A10. Smoking exacerbates vascular complications due to diabetes. | - 0.45 ± 0.74 | - 0.19 ± 0.55 | - 0.35 ± 0.68 |
| Total attitude score (0-100) | 43.40 ± 8.66 | 45.83 ± 7.60 | 44.27 ± 8.35 |

1. **Table 3.** Description of Patients’ practice Score in Detail

| **Practice** | **Male** | **Female** | **Total Score** |
| --- | --- | --- | --- |
| P1. When was your last ophthalmologist referral? | 0.76 ± 0.42 | 0.71 ± 0.45 | 0.74 ± 0.43 |
| P2. Would you use herbal medications for controlling diabetes? | 0.72 ± 0.45 | 0.50 ± 0.50 | 0.64 ± 0.48 |
| P3. When was your last nutritionist referral? | 0.04 ± 0.19 | 0.02 ± 0.15 | 0.03 ± 0.18 |
| P4. How many times a week do you examine your feet? | 0.41 ± 0.49 | 0.19 ± 0.39 | 0.33 ± 0.47 |
| P5. Have you glucometer? | 0.96 ± 0.19 | 0.92 ± 0.26 | 0.94 ± 0.22 |
| P6. When is proper time to check blood glucose by glucometer? | 0.72 ± 0.45 | 0.52 ± 0.50 | 0.64 ± 0.47 |
| P7. How many days a week do you exercise? | 0.12 ± 0.32 | 0.02 ± 0.15 | 0.08 ± 0.28 |
| P8. How many main meals do you have daily? | 0.72 ± 0.45 | 0.61 ±0.49 | 0.68 ± 0.46 |
| P9. Last year, how many times did you visit a doctor? | 0.13 ± 0.34 | 0.11 ± 0.32 | 0.12 ± 0.33 |
| P10. Do you smoke? | 0.54 ± 0.50 | 1.00 ± 00 | 0.70 ± 0.45 |
| P11. Have you ever participated in a diabetes education class? | 0.00 ± 0.00 | 0.00 ± 0.00 | 0.00 ± 0.00 |
| Total practice score (0-100) | 46.66 ± 13.80 | 42.20 ± 10.59 | 45.06 ± 12.87 |

1. **Table 4.** Description of Patients’ self-care Score in Detail

| **Self-care** | **Score (0-100)** |
| --- | --- |
| Diet | 56.92 ± 9.76 |
| Exercise | 9.82 ± 14.73 |
| BS Glucometer | 27.65 ± 29.93 |
| Foot Care | 40.07 ± 20.76 |
| Medications | 79.48 ± 40.55 |
| Smoking | 92.30 ± 26.76 |
| **Total self-care scores :** | **46.21 ± 10.23** |
